# Introduction to the editorial "Development of a syllabus for postgraduate respiratory physiotherapy education: the HERMES Respiratory Physiotherapy project" (Força-tarefa da European Respiratory Society para harmonização da educação na especialidade de Fisioterapia Respiratória: uma iniciativa de abrangência mundial)

**DOI:** 10.1590/bjpt-rbf.2014.0094

**Published:** 2015

**Authors:** Fabio Pitta

**Affiliations:** Laboratório de Pesquisa em Fisioterapia Pulmonar (LFIP), Departamento de Fisioterapia, Universidade Estadual de Londrina (UEL), Londrina, PR, Brasil

Dear colleagues,

This edition of the Brazilian Journal of Physical Therapy (BJPT) features an editorial concerning an important initiative to harmonize Graduate education in Respiratory Physiotherapy throughout the world. This editorial is about the work of a task force organized by the European Respiratory Society (ERS) which aims at standardizing the essential content to be reached in the area of Respiratory Physical Therapy, regardless of the country in which the Physical Therapist is working on. More than 150 well known experts in Respiratory Physical Therapy from all over the world contributed to this initiative. Moreover, since the goal of the task force is to disseminate this information in order to reach the widest possible range of professionals, this knowledge has been forwarded to the most important national scientific vehicles of Physical Therapy, such as the Brazilian Journal of Physical Therapy (BJPT) in the case of Brazil. Since the idea is to "spread the word" for the largest number of professionals and languages, this is the reason why this editorial, published in the BJPT, is in Portuguese, as opposed to the typical content, which is currently published in English by the journal. The editorial includes links to the most relevant available documents already developed by the task force. We hope the content is useful to as many Physical Therapists as possible!

With kind regards

Fabio Pitta

## EDITORIAL

### Força-tarefa da European Respiratory Society para harmonização da educação na especialidade de Fisioterapia Respiratória: uma iniciativa de abrangência mundial

De forma geral, a educação em Fisioterapia é marcadamente heterogênea em todo o mundo. Similarmente, o nível de formação também é extremamente heterogêneo em escala mundial nas diferentes subáreas de especialização da profissão, como a Fisioterapia Respiratória. O Brasil, por exemplo, tem, há alguns anos, o privilégio de ter provas de título de especialista em Fisioterapia Respiratória e em Fisioterapia em Terapia Intensiva organizadas pela Associação Brasileira de Fisioterapia Cardiorrespiratória e Fisioterapia em Terapia Intensiva (ASSOBRAFIR); no entanto, a grande maioria dos países não possui tal certificação oficialmente formalizada. Isso dificulta o intercâmbio profissional entre fisioterapeutas respiratórios de diferentes regiões do mundo, assim como, muitas vezes, torna difícil para pacientes e outros profissionais e gestores da área da saúde saber que nível de conhecimento esperar de um fisioterapeuta especializado na área respiratória. Adicionalmente, é muitas vezes complexo engajar fisioterapeutas respiratórios em tarefas mais especializadas ou avançadas, visto que a *expertise* de um especialista nessa área pode variar consideravelmente, tanto em nível internacional como nacional e até mesmo regional, dependendo do local de treinamento (especialização). Assim como nas outras áreas da Fisioterapia, isso pode fazer com que a especialidade se mantenha em um nível mais baixo do que o ideal, limitando a prática da Fisioterapia a uma realidade abaixo do que um fisioterapeuta bem treinado poderia atingir.

Portanto, é no melhor interesse de fisioterapeutas, pacientes e outros profissionais e gestores da área da saúde que um conjunto minimamente padronizado de habilidades avançadas para um especialista em Fisioterapia Respiratória esteja disponível, conjunto esse que deve ser amplamente aceito por *experts* nessa área. Com base em evidências científicas, espera-se que um fisioterapeuta respiratório seja uma das peças fundamentais no cuidado de pacientes de todas as idades com doenças respiratórias agudas e crônicas.

Um grupo de trabalho da *European Respiratory Society* (ERS, ou Sociedade Respiratória Europeia) denominado Força-tarefa ERS-HERMES Fisioterapia Respiratória, com a contribuição de outros participantes importantes, como a *European Respiratory Care Association* (ERCA), a ASSOBRAFIR, outras entidades nacionais do mundo todo e *experts* com reconhecimento internacional na área de Fisioterapia Respiratória, trabalhou para identificar qual conteúdo deve ser considerado essencial em um programa de treinamento da especialidade de Fisioterapia Respiratória^1^. Após um sistemático e rigoroso processo de consulta a *experts* do mundo todo, esse conteúdo essencial foi definido e agrupado em dois módulos: um para fisioterapeutas envolvidos no tratamento de pacientes adultos e outro para os envolvidos no tratamento de pacientes pediátricos. Módulos opcionais foram desenvolvidos para fisioterapeutas envolvidos no tratamento de pacientes em estado crítico na unidade de terapia intensiva, tanto adulta quanto pediátrica. Esses módulos cobrem aspectos e conteúdos da Fisioterapia Respiratória que atingiram um nível muito alto de consenso entre mais de 150 *experts* do mundo todo. Os módulos estão agora disponíveis *on-line* em língua inglesa no link http://ow.ly/zYkOD junto com um artigo (também publicado em língua inglesa na revista *Breathe* ^2)^ que descreve o meticuloso caminho trilhado pela Força-tarefa até finalizar esse processo, disponível no seguinte link: http://breathe.ersjournals.com/content/10/3/220.full

A Força-tarefa ERS-HERMES Fisioterapia Respiratória acredita que esse é um modelo para todos os educadores envolvidos em Fisioterapia Respiratória utilizarem como uma base para homogeneizar conteúdos educacionais, independente do país onde trabalhem. O conhecimento e as habilidades apresentados também permitem a fisioterapeutas em especialização na área respiratória uma visão geral do nível esperado de prática internacional baseado na opinião de *experts* em Fisioterapia Respiratória de várias partes do mundo: http://hermes.ersnet.org/images/Physio_HERMES_Delphi_3_Analysis_report_-_Copy.pdf

Atualmente está em desenvolvimento pela Força-tarefa um currículo detalhado, mapeando como esse conteúdo pode ser ensinado e incluindo desfechos de aprendizado, assim como métodos de avaliação, atividades de ensino e exposição mínima clínica/educacional. Esse trabalho deverá ser finalizado e amplamente divulgado mundialmente em breve.

Espera-se que, ao se determinar e respeitar um conteúdo essencial minimamente padronizado nessa especialidade, assim como programas de treinamento e certificação internacional, fisioterapeutas respiratórios terão a possibilidade de se certificar com mais segurança de acordo com os padrões do ERS-HERMES Fisioterapia Respiratória e, portanto, terão uma possibilidade mais real de cruzar profissionalmente fronteiras internacionais. Em outro nível, essas iniciativas possibilitarão a gestores ter uma ferramenta imparcial e de qualidade valiosa para credenciar legalmente profissionais que atingem um nível de competência adequada. O objetivo final dessa Força-tarefa é preparar o especialista em Fisioterapia Respiratória para a entrada na prática clínica de forma independente, porém consciente do papel que exerce em uma equipe de cuidado profissional, oferecendo o melhor nível possível de atendimento para pacientes com doenças respiratórias.

## References

[1] Troosters T, Pitta F, Oberwaldner B, Lewko A, Inal-Ince D, Grant K (2015). Development of a syllabus for postgraduate respiratory
physiotherapy education: the Respiratory Physiotherapy HERMES
project. Eur Respir J.

[2] Pitta F, Mitchell S, Chatwin M, Clini EM, Emtner M, Gosselink R (2014). A Core syllabus for post-graduate training in
respiratory physiotherapy. Breathe.

